# Robust Response to *Plum pox virus* Infection via Plant Biotechnology

**DOI:** 10.3390/genes12060816

**Published:** 2021-05-27

**Authors:** Michel Ravelonandro, Pascal Briard, Ralph Scorza, Ann Callahan, Ioan Zagrai, Jiban K. Kundu, Chris Dardick

**Affiliations:** 1 UMR-BFP-1332, INRAE-Bordeaux, Bordeaux-UniversityII, 71 Avenue Bourleaux, 33883 Villenave d’Ornon, France; pascal.briard@inrae.fr; 2 USDA-ARS Fruit Station, 2217 Wiltshire Road, Kearneysville, WV 25430, USA; ralph.scorza@gmail.com (R.S.); ann.callahan@usda.gov (A.C.); Chris.Dardick@usda.gov (C.D.); 3 Fruit Research and Development Station Bistrita, Drumul Dumitrei Nou street, 420127 Bistrita, Romania; izagrai@yahoo.com; 4 Crop Research Institute, Drnovska 507/73, 161 06 Praha, Czech Republic; jiban@vurv.cz

**Keywords:** RNAi, hairpin, gene construct, *Plum pox virus*, *Prunus domestica*, resistance

## Abstract

Our goal was to target silencing of the *Plum pox virus* coat protein (*PPV* CP) gene independently expressed in plants. Clone C-2 is a transgenic plum expressing CP. We introduced and verified, in planta, the effects of the inverse repeat of CP sequence split by a hairpin (IRSH) that was characterized in the HoneySweet plum. The IRSH construct was driven by two CaMV35S promoter sequences flanking the CP sequence and had been introduced into C1738 plum. To determine if this structure was enough to induce silencing, cross-hybridization was made with the C1738 clone and the CP expressing but *PPV*-susceptible C2 clone. In total, 4 out of 63 clones were silenced. While introduction of the IRSH is reduced due to the heterozygous character in C1738 plum, the silencing induced by the IRSH *PPV* CP is robust. Extensive studies, in greenhouse containment, demonstrated that the genetic resource of C1738 clone can silence the CP production. In addition, these were verified through the virus transgene pyramiding in the BO70146 BlueByrd cv. plum that successfully produced resistant BlueByrd BO70146 × C1738 (HybC1738) hybrid plums.

## 1. Introduction

Genetic engineering in plants is an accurate technology aiming at the introduction of a foreign sequences into the genome. In order to overcome the incoming viral genome, the present technology consists of protecting plants against virus [1] through a pre-existing gene silencing approach. The challenging example is the *PPV* that devastatingly infects many *Prunus* genera [2]. Genetically engineering *Prunus domestica* with a constitutively expressed coat protein gene (CP) from *PPV* resulted in lines that highly expressed CP to those that do not express CP gene [3]. One of the two lines that do not transcribe CP RNA nor accumulate CP, is resistant (C5 clone) and the other susceptible (C6 clone) [3,4]. C-6 plum which harbors the full-length CP gene construct, shows some alteration in the CaMV35S promoter and the GUS cassette. This clone has been shown to be highly susceptible to *PPV* with no evidence of a silencing mechanism [4]. C5 clone, now known as HoneySweet is the sole clone resistant to *PPV* infection [3,4]. It is not just the absence of expression while the gene is present as in C6 plum that makes the plant resistant to *PPV*. It must be something about the structure of the insertion event. A high diversity of research was developed to better understand the relationship between the virus sequence transgene introduced in the HoneySweet plum and the high level of resistance phenotype of the clone [5,6,7]. The recent publication of the whole genome sequencing of plum and the insertion events of HoneySweet [8] demonstrated that a multiple viral transgene copy has been introduced into the plum genome. These new findings based on these results clarified remarkably the relationship between the number of transgene copy and virus resistance. One of the two insertion events in HoneySweet is designated “insertion event 2” [8] which consists of two inverted repeats of the *PPV* CP gene split by a hairpin and is potentially the key to the resistance. The 132 bp of the 3′untranslated sequence and unpaired in the duplication of the *PPV* sequence, reshapes the hairpin and together they represent the inverse repeat of CP sequence split by a hairpin (IRSH) gene construct responsible for the *PPV* resistance [8,9].

Scorza et al. have reengineered it in the BlueByrd (BO70146) [10] plum and have also successfully confirmed through the majority of clones obtained that they were resistant to *PPV* infection [9] in a two-year greenhouse experiment. In line with silencing [11] as the regulatory phenomenon related to this IRSH *PPV* CP, extracted from the HoneySweet plum, we wanted to explore here the regulatory phenomenon. By combining the virus transgene resource of the C1738 plum harboring one copy of the IRSH *PPV* CP and a NPTII marker gene with that of the C2 clone encoding CP within two gene markers, GUS and NPTII [3]. Epigenetics is among a eukaryotic process that is not deeply investigated in woody plants [12]. Although the approach based onto the gene regulation related to the plant development has been exploited [13]. The reported data about the transgene flow from HoneySweet indicated, excepted the species criteria, that in theory, there is no any special barriers about the natural cross between *Prunus* species [14]. Undoubtedly, the facets of epigenetics are not negligible, when depicted as an uncontrolled variation of genes that express under the pressure of diverse types of environmental factors (abiotic or biotic stress, growth inhibition, etc.) [15].

For the above reasons, controlled studies based onto the assessment of the genetic and phenotypic differences related to the structure and the gene for gene interference of these viral genes were examined here [3,9]. Directed cross hybridization between the two clones, C2 and C1738 clones, was attempted in order to study the silencing mechanisms occurring and to decipher the epigenetic phenomenon in action, as well as developing tools and methodology to understand the genetic involvement of the *PPV* CP sequences. Four hybrid seedlings harboring both the encoding transgene CP and the IRSH construct were selected. Similar to the transgenic Honeysweet plum, the IRSH harbored by the four hybrid plums silenced the encoding CP gene. Another hybridization with the conventional BlueByrd (BO70146) plum allowed us to demonstrate that the IRSH without the intact CP generates hybrid resistant clones. Taken together these results, the serendipitous hairpin CP structure discovered in *Prunus* represents, first an original genetic tool to better understand the epigenetic phenomenon in perennial trees, and secondly, it reflects a sustainable source of resistance gene to *PPV* infection.

## 2. Materials and Methods

### 2.1. BlueByrd (BO70146) Plum, GF-305 Peach and Virus Resistance

BlueByrd cv.plum, and GF-305 peach were used in high containment greenhouse assays as positive controls for *PPV* infection. In order to test plant resistance, each clone was, first, propagated onto the susceptible rootstock *Prunus marianna* (GF8.1) in a high containment greenhouse (agreement for the use of genetically modified organisms, GMO, for research and development, number 2000, 28 October 2015, Ministry of Education and Research). Since an available number of replicates (3–6 copies) was obtained, plants were graft-inoculated prior to their transfer in cold for setting up an artificial dormancy. *PPV*-M was chosen to infect the clones because it causes more severe disease [4,5,6,16,17]. Initial testing for infection was based on experimental evidence for *PPV* infection through the appearance of symptoms (mosaic on BlueByrd plum and typical leaf distortion on peach) from 4 weeks after the first bud-break. In light of the virus spread in scions, DAS-ELISA was also used to ensure that the tested trees were successfully infected by the challenger *PPV* [18,19]. Specific polyclonal antibodies raised to *PPV* (LCA, La Rochelle, France) were used according to the manufacturer’s recommendations. All assays were validated at the same time with infected GF.8.1 rootstocks. Infection was both recorded through symptom evaluation and an OD value from the DAS-ELISA higher than 0.1 (OD value read at 405 nm using phosphate buffered saline-Tween as the background value). Following the third cycle of dormancy, molecular detection with RT-PCR confirmed the infection status. OneStep RT-PCR was used to detect *PPV* RNA. 1 μg of total RNA was used in a reagent mixture of 50 μL containing buffer, dNTPs, 1U of mixed enzyme (RTase, Taq DNA polymerase) (Qiagen-Kit, Valencia, Hilden, Germany) and 1 μL of the following primers, YGAP (YGAKGABATGTACATTCC) and RB8740 (TCCGGATCCGTTGTTGCTGGMGTGAAAATGGGGTTG) according to [16]. The reaction consisted of an incubation of 30 min at 50 °C followed by a denaturing step at 95 °C during 15 min. PCR was performed with 40 cycles of denaturing at 95 °C for 30 s, 50 °C for 30 s, 72 °C for 1 min and a final extension at 72 °C, for 10 min. Because the IRSH construct does not span to the Nib (Nuclear Inclusion b) cistron of *PPV* RNA, separate PCR reaction forming an amplicon of 460 bp spanning the COOH part of the *PPV*-Nib cistron and NH2 of the CP gene was used to detect the ongoing spread of *PPV*.

### 2.2. Transformed Plums C1738

**Figure 1:**
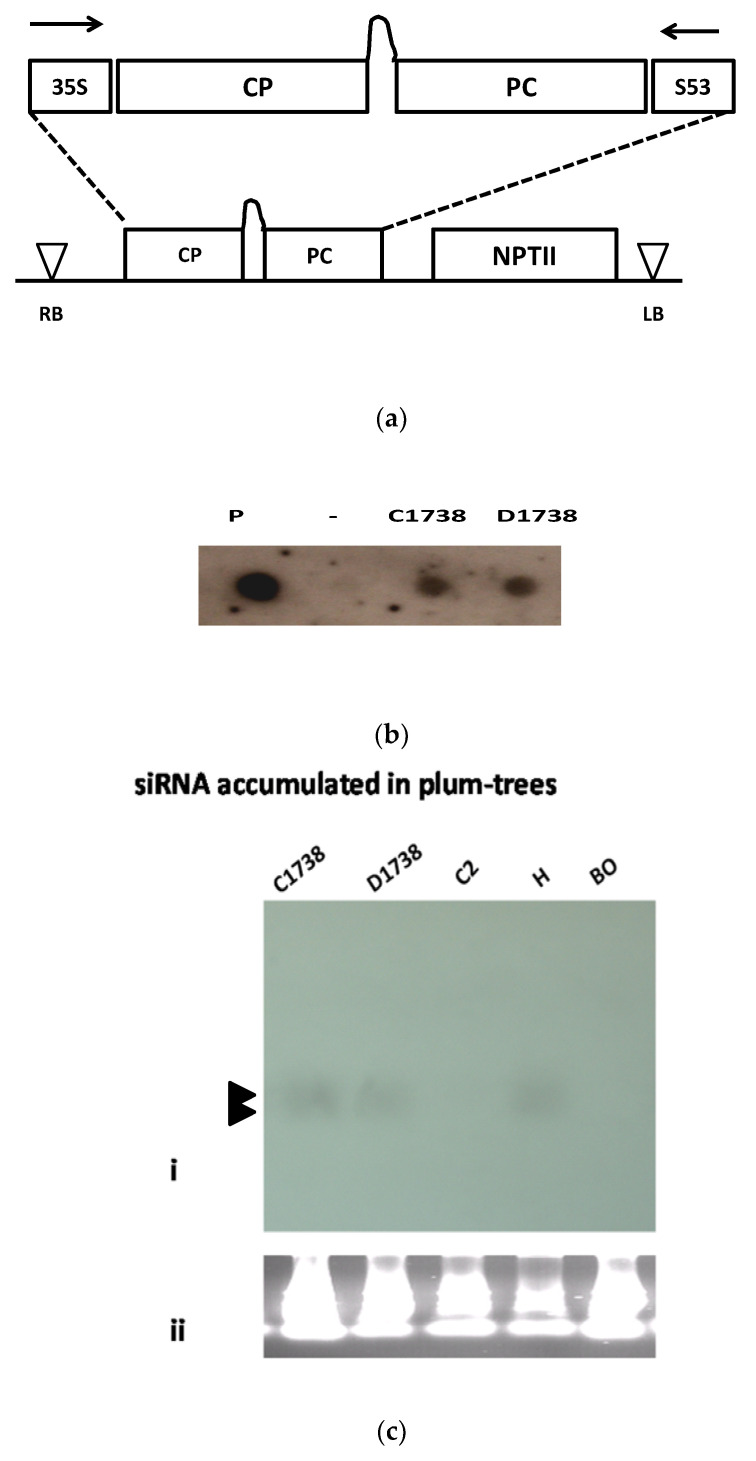

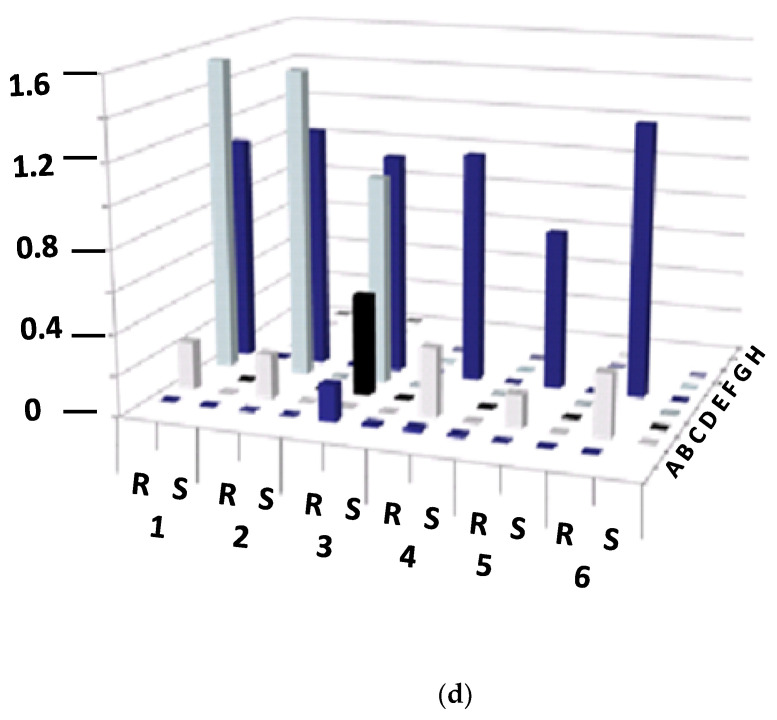
*PPV* resistance of C1738 plum. (**a**) Schematic diagram of the T-DNA of the recombinant pBINPLUS/ARS-IRSH construct (RB: Right border, LB: left border). (**b**) Dot-blotting of plant DNA for checking the homologous copy number of C1738 and D1738 plums. P, recombinant PPV CP plasmid, (-) no DNA, C: C1738 clone, D: D1738. clone. (**c**) (i) Northern blot analysis of the siRNA accumulated in the non-infected plums including C1738, D1738, C2, H: HoneySweet and BO: BlueByrd plums. Arrowheads in the left margin indicate the siRNA doublet (21 and 24 nt) bands detected with the labeled α ^32^P dCTP-*PPV* CP amplicon probe. (ii) Ribosomal RNA control. (**d**) Behavior of the C1738 clone over 4 dormancy cycles: Histograms representing the average of different OD values of DAS-ELISA tests (scale bar) from leaves sampled from 6 plant replicates at different dates A raw: 25 July 2012, first dormancy cycle, B raw: 4 April 2013, second dormancy cycle C: 14 May 2013, D raw: 4 November 2014, third dormancy cycle, E raw: 17 September 2015, fourth dormancy cycle, F: 12 January 2016, G raw: 20 July 2016, fifth dormancy cycle, H: 4 October 2016 following to the bud-breaking date (A, B, D, E and G raws) (indicated at the right). Printed R, leaves collected from the GF-8.1 rootstocks, and S, those from the transgenic shoots.

The IRSH construct was isolated from the resistant HoneySweet plum as previously described [9]. It had been cloned into the pBINPLUS/ARS vector (Figure 1a), and hypocotyl slices of BlueByrd (BO70146) cv plum [10] were co-inoculated with the *Agrobacterium tumefaciens* containing the construct resulting in the C1738 plum used in this study [9].

### 2.3. Hybridization

All respective male progenitors were hand-emasculated. Over the next 2–3 days, pollen was applied on pistils with a brush. Two trials of hybridization were performed, first C2 × C1738 and C1738 × C2 and second BlueByrd × C1738 and C1738 × BlueByrd.

### 2.4. Hybrid Selection via GUS Assays

Young leaves from hybrid clones were cut and shaped in a small square that were introduced in an eppendorf tube of 1.5 mL. They were soaked in 500 µL of 50 mM Na_2_HPO_4_, pH 7.0 and 0.1% Triton X-100 containing the chromogenic X-Gluc (5-bromo-4-choloro-3-indolyl) β-D-glucuronic acid substrate, overnight at 37 °C. After pipetting the substrate, leaves were bleached by washing with 70% ethanol that led to the fixation of the blue color revealing the positive assay of GUS [20].

### 2.5. Methylation of Transgene

Plant genomic DNA of studied clones were extracted according to [16,17,21]. In total, 2 μg of DNA were digested overnight in parallel, with *BfuCI* and the isoschizomer *MboI* at 37 °C. After a precontrol of the digestion efficiency, one aliquote (1/10) of the digested DNA was amplified by PCR using the couple of primers 340 Fw and 660 Rev according to [16,17,22]. PCR conditions were one cycle of 94 °C, 2 min, 40 cycles of 94 °C, 30 s, 55 °C, 30 s, 72 °C, 1 min, followed by one cycle of 72 °C for 10 min prior to stop at 12 °C. In total, 1 Kb DNA weight marker (Invitrogen, Thermo Fisher Scientific, Waltham, MA, USA), an aliquot of the amplified DNA was fractionated onto a 2.5% agarose gel electrophoresis. The occurrence of an amplicon of 425 bp symbolized that the template is methylated.

### 2.6. siRNA Detection

Total RNAs were extracted according to [16,17,22,23]. 30 μg of the total RNA were loaded on 16% of a denaturing urea-PAGE. Electrophoresis was carried out at 25 mA with 0.5 TBE. during 6hours. Following to an electroblotting transfer with 0.5 TBE, in cold (at 4 °C) onto the NX membrane, (GE Healthcare, Buckinghamshire, UK), siRNAs were probed with a labeled α ^32^P dCTP-*PPV* CP amplicon probe.

### 2.7. Production of a Labeled ^32^P PPV CP Probe

To detect the *PPV* CP sequence either introduced or transcribed in plum genome, the use of a ^32^P molecular probe is among the specific and reproducible system [16,17]. By PCR-amplifying the *PPV* CP sequence, we used, as template, the pGA482GG/PPVCP-33 recombinant plasmid [3] in a reagent mixture of 50 μL containing buffer, dATP, dGTP, dTTP and, α ^32^P dCTP (Perkin Elmer, Waltham, MA, USA), 1U Taq DNA polymerase (Qiagen-Kit, Valencia, Hilden, Germany) with forward primer (CPFwd: AAGCTGACGAAAGACAGGACGAG) and reverse primer (RevCP: CTACACTCCCCTCACACCGAGGAA). The conditions of the PCR were as follows: denaturation at 94 °C, for 2 min, 40 cycles of denaturaion at 94 °C for 1min, annealing at 55 °C for 1 min, amplification at 72 °C for 1 min and final extension at 72 °C for 10 min. Prior to use, the labeled ^32^P *PPV* CP probe was purified through a size-exclusion MicroSpin G25 column (Amersham, GE Healthcare, Buckinghamshire, UK).

### 2.8. Western-Blotting Assays

In order to serologically detect any viral protein, the high specificity of the antisera is among the determinant criterion. Specific antisera to *PPV* were produced from the intra-muscular injection of purified *PPV* to rabbits (INRAe-Bordeaux). To perform the detection of the *PPV* CP in plum tissue, total soluble proteins were extracted from young leaves in a lysis buffer according to [4]. 300 μg of total soluble proteins were fractionated by 12% SDS-Polyacrylamide gel electrophoresis (PAGE). Proteins were electrotransferred onto a nitrocellulose membrane blot and probed with a rabbit polyclonal antibody against *PPV* (INRAe-Bordeaux) [4]. The reaction was revealed with anti-rabbit (goat) secondary antibodies coupled to phosphatase alkaline (goat anti-rabbit, Jackson, ImmunoResearch, West Grove, PA, USA). Chromogenic immunodetection was done with NBT/BCIP colored substrate (Sigma Aldrich, Saint-Louis, MI, USA). The expected band, a protein of 36 KDa was followed with a pre-stained molecular weight marker (Invitrogen, Gaithersburg, MD, USA).

## 3. Results

### 3.1. Transgenic C1738 and D1738 Clones

Two clones containing the IRSH construct (Figure 1a) previously characterized by Scorza et al. [9] C1738 and D1738 clone, were further characterized. D1738 plum is among the 18 clones characterized by Scorza et al. [9]. It had been shown to be resistant [9] and is used a reference. However, the development of the C1738 clone, likely harboring one transgene copy (Figure 1b), was delayed. Similarly, to the HoneySweet plum, all clones harboring the IRSH were expected to accumulate siRNA related to the CP (Figure 1c). In order to assess the behavior of this clone to *PPV* infection, C1738 clone was replicated by grafting onto the *P. marianna* GF 8.1 rootstocks. Six plant replicates were challenged to *PPV* infection. PPV detection in rootstocks was crucial for indicating that scions are infected, At least three to four dormancy cycles were regarded as reliable to record the *PPV* spread in whole plants [4,16,17]. Once *PPV* moved from the rootstock to the scion [4,7,16,17,18,19], *PPV* is detectable in any susceptible hosts, from the fourth week following to the bud-break of the first dormancy cycle. From the fourth cycle of dormancy (Figure 1d: E raw: 17 September 2015), infected rootstocks differed from the resistant scions. Through either the appearance of symptoms in rootstocks (not shown) or/and the analytical detection of *PPV* carried out in laboratory, positive DAS-ELISA in the rootstocks, ensured that the tested trees were under pressure from the challenger *PPV* (Figure 1d) [4,7,16,17,18,22,23]. Histograms that represent the relative levels of *PPV* infection in the rootstock section are opposed to those of the C1738 scions. Not one C1738 plant was infected as indicated by DAS-ELISA readings. These studies were confirmed after carrying out total RNA extraction and RT/PCR analyses. Interestingly, no *PPV* RNA was detected in the C1738 scion (not shown). These results suggested that like D1738, C1738 is highly resistant plant to *PPV* infection (Figure 1d).

### 3.2. Cross Hybridization between the Hexaploid C-2 and C-1738 Clones

**Figure 2:**
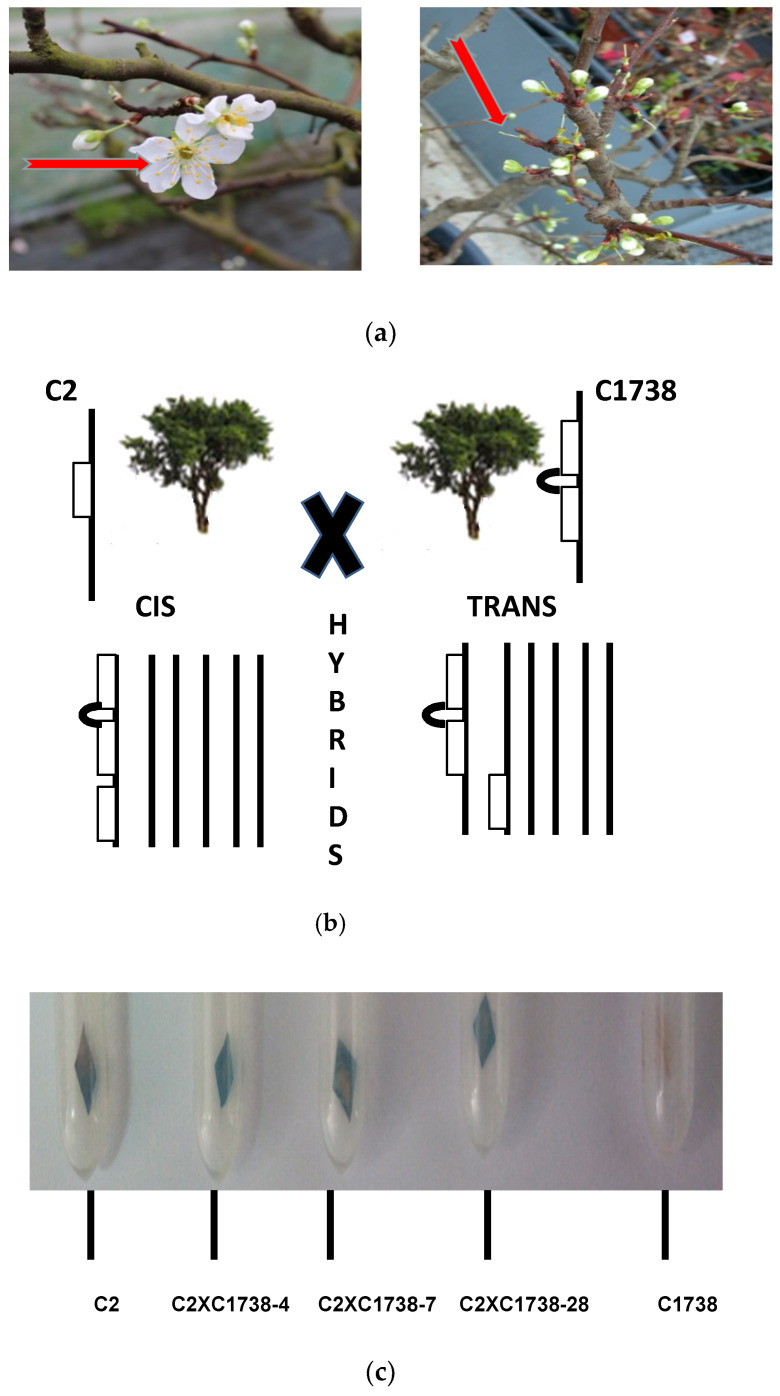
Hybridization of C2 and C1738. (**a**) From left to right: intact and emasculated flowers of clone C1738; (**b**) Scheme of the cross-hybridization between C2 and C1738 clones; (**c**) Histochemical analysis of GUS activity in small leaves sampled from the two parental clones, C2 and C1738 and 3 C2 × C1738 hybrids.

To understand the silencing mechanisms related with the IRSH construct, C1738 plum and C2, the plum clone harboring the expressed transgene CP, were hybridized. Following to the prerequisite emasculation, respectively, of the flowers of C-2 clone and those of C-1738 plum (Figure 2a) for reciprocal crosses, dried and lyophilized pollens from each of the clones were, respectively, applied onto the pistils. More than 70 fruit were collected from the C2 × C1738 hybridization, however less than 10 fruit were obtained with C1738 × C2 (Table 1). Embryos were pre-incubated in the cold room prior to their move to growth chamber. Following to the acclimation of rooted seedlings in greenhouse, leaves were collected and were tested for GUS [20] indicating the presence of the expressed transgene CP initially introduced in C2 clone. A few hybrids did not develop, so Table 1 summarizes the results about 63 plants. As example, Figure 2b shows that the genetic cross C2 × C1738 gave 32/60 transgenic hybrid clones. A ratio that matches to the Mendelian rules as we have already observed with cross hybridization between “HoneySweet” plum and, respectively, the conventional plums, Ente 303 and Quetsche 2906 [24]. Only one of the 3 hybrid clones from the cross hybridization with C1738 × C2.was positive for GUS expression.

### 3.3. Inhibition of the CP Gene Expressed in C-2 Clone

#### 3.3.1. Heritable Epigenetics

**Figure 3:**
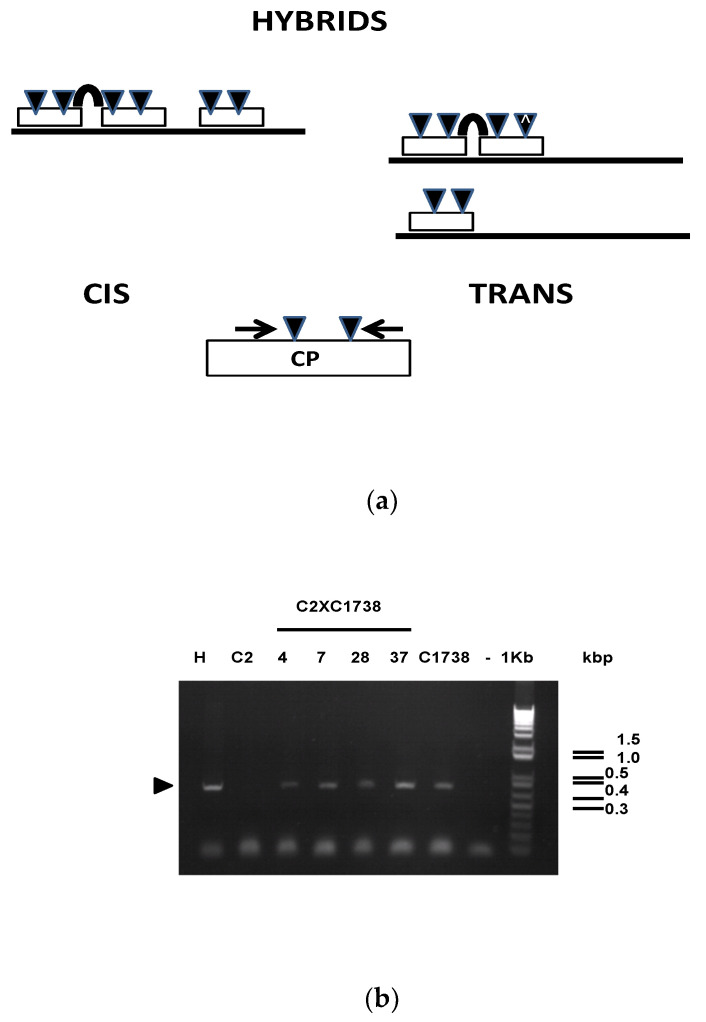

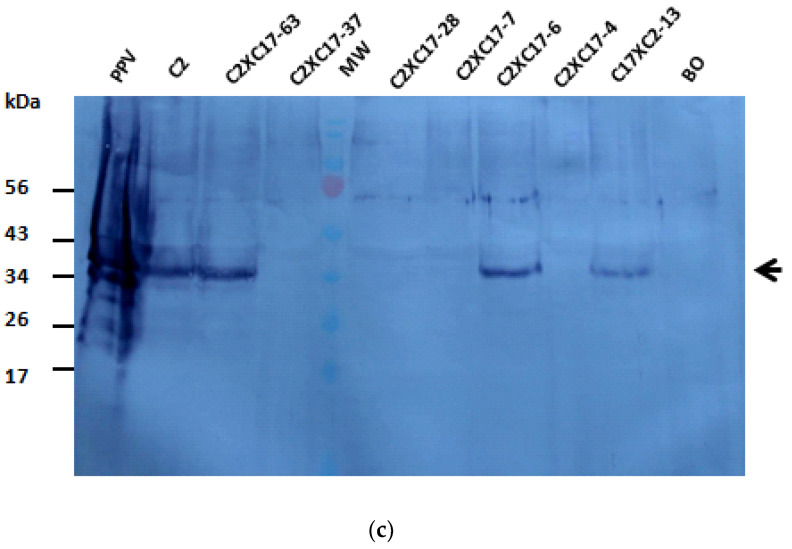
(**a**) Scheme of the *PPV* CP transgene integrated either in Cis- and Trans-position in hybrid plums: Vertical arrowheads indicate the position of the targeted GATC restriction sites flanked by the primer pair, 340 Fwd and 660 Rev (arrows) used in PCR reaction. (**b**) Agarose gel analysis (2.5%) of amplicon (arrowhead in the left margin) from the over digested DNA of the different plants, from left to right, lanes: H, HoneySweet plum (as positive control), C2: cloneC2, C2 × C1738 hybrid clones (4, 7, 28, 37), C1738, (-), no DNA, 1 kb markers (Invitrogen, Gaithersburg, MD, USA). (**c**) Immunoblotting of total protein extracts from plum leaves (left to right): *PPV*-infected Bluebyrd BO, used as positive control, C2, C2 × C1738 63, C2 × C1738-37 hybrids, pre-stained MW (Invitrogen, Gaithersburg, MD, USA), C2 × C1738-28, C2 × C1738-7, C2 × C1738-6, C2 × C1738-4, C1738 × C2-13 hybrids and virus-free Bluebyrd BO, used as negative control. Arrow in the right margin represents the expected *PPV* CP.

In line with the regulatory mechanism related with epigenetics such as methylation [7,16,17,22,23,25], molecular analysis of the transgene CP from the 33 hybrid clones was studied (Table 1). Leaves were collected and the plant DNA extracted. A coupling reaction related to the over-digestion of the genomic DNA with *BFuCI* restriction enzyme that can cut at the 2 GATC sites of the CP transgene only if the sites are not methylated. If the sites are methylated then by the subsequent PCR-reaction using two primers flanking the 2 sites, an amplicon of 425 bp is present. Surprisingly, only 4/33 clones that express GUS (Figure 2b) showed a methylated transgene (Figure 3b).

To conclude, from the genetic cross C2 × C1738, 28/60 of hybrid clones had no evidence of methylation indicative of silencing, 32 clones harbor the transgene CP of C2 clone and amidst the 32 clones only four clones have evidence of methylation and probably harbor the two transgenic events (Table 1). The inheritance of methylation has frequently occurred in eukaryotic systems, and these viral origin genes are no different [12,13,16,17,22,24]. It appears to be methylated regardless of whether or not the insertion of the viral genes in one locus (cis position) or their split in two different loci (trans position), where four hybrid clones were identified; C-2 × C-1738-4, -7, -28 and -37 hybrids (Figure 3). To conclude, from the genetic cross C2 × C1738, 28/60 of hybrid clones were not methylated and probably did not carry the IRSH, 28 clones harbor the transgene CP of C2 clone and only four clones harbor the two targeted events (Table 1).

#### 3.3.2. Western-Blotting Assays

In order to confirm the potential silencing of the CP resulting in lowered expression by the four clones, that are both positive for GUS and the methylated transgene, a protein study to determine if the *PPV* CP gene was affected. This would then measure the targeted phenomenon confirming the efficient of the IRSH CP gene cassette to silence the CP gene belonging to the parental C-2 clone. Total soluble proteins from different clones including the four C-2 × C-1738-4, -7, -28 and -37 hybrids were assayed through western-blotting experiments. Figure 3c shows that these four clones possessing the methylated transgene do not accumulate CP. In parallel, hybrid clones used as control, known as harboring an unmethylated CP gene, C-2 × C-1738-6 and -63, chosen as similar control to the parental C2 clone both express the CP gene [3]. Similarly, the sole C-1738 × C2-13 hybrid clone, that showed a positive GUS assay and harbors an unmethylated DNA, was confirmed by the immunoblot had detectable CP. Silencing was based on the methylation mechanism impacted by the modified transcription of the *PPV* CP gene originated from clone C2. There is evidence here that the RNA-based silencing results from the homologous methylated gene co-integrated in planta.

### 3.4. Inheritability of PPV Resistance in Hybrid Clones of BO146xC-1738 Clone

**Figure 4:**
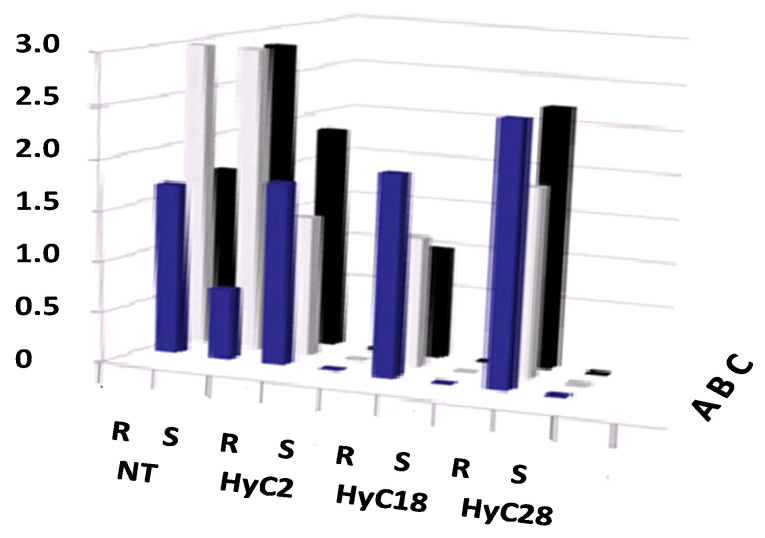
Histograms representing the average of the different OD values of DAS-ELISA tests (scale bar) from leaves sampled from 5 plant replicates performed at different bud-breaking period, blue: A, cycle 1, grey: B, cycle 2 and black: C, cycle 3. NT, non-transformed BlueByrd BO70146 and BlueByrd BO70146 × C1738 hybrids including HybC1738-2 (HyC2), -18 (HyC18), 28 (HyC28). R, graphs of leaves sampled from the *P. marianna* GF-8.1 rootstocks, and S, those from the transgenic shoots.

As previously shown, cross-hybridization enables the IRSH transfer. Since the identification of the hybrid clones, the occurrence of the methylated transgene was verified. Through the cross-hybridization with the conventional Bluebyrd, already characterized as highly susceptible to *PPV* [4,7,15,16,17,18,19,23], a lot of hybrid clones were obtained (11/30) of the faster growing clones were propagated onto *P. marianna* GF 8.1 rootstock. Figure 4 shows the relevant efficiency of IRSH as measured by DAS-ELISA values following infection.

All three hybrids tested including Bluebyrd BO70146 × C1738-2 (HyC2), -18 (HyC18), -28 (HyC28) behaved similarly to the parental C1738 plum ((Figure 1d). There is an evidence that, the transgenic scion (S) had negligible value compared to the relative amount of *PPV* in non-transgenic Bluebyrd BO70146 (NT) and susceptible *P. marianna* GF-8.1 rootstock (R), shown to be susceptible from the first dormancy cycle (blue bar graphs in A raw). To gain an understanding of the plant phenotypes, the occurrence solely of symptoms in control (NT plants and rootstocks, R) (not shown) allows the confirmation of diseased trees. Here we present the data of the serological assays depicting the challenging assays to *PPV* infection following three cycles of dormancy (A, B and C raws) reflecting the natural time for increased viral loads. Based on the sampling of leaves of tested plants including the conventional BO70146 plum (NT) and the different replicates of the three selected hybrid clones (HyC2, HyC18 and HyC28), the histograms represent an average OD values of the five plant replicates of each clone. Based on the homologous data of the serological studies, these selected hybrid clones are highly resistant. Genetic transfer of the IRSH construct has been successfully established. Expectedly, RNAi produced from the dsRNA transcribed from the two CaMV35S promoter can be inherited from one locus and expressed as a resistance trait such as a haploid parent, similar to the HoneySweet plum, source of the IRSH construct [5,6,8,9,23].

## 4. Discussion

These studies showed that the bidirectional promoters flanking the two inverted repeats of *PPV* CP gene functioned in the new background as they had in the original HoneySweet [8,9]. The IRSH structure was efficiently transcribed by the RNA polymerase II in nuclei [26] prior to the sequential transfer of the silencing in cytoplasm. Epigenetics is among eukaryotic processes that regulate plant development (growth, flowering, fruit development etc.) as well as under environmental pressure [12,13,14,18,19,25]. Briefly, cell differentiation is associated with phenotypic changes [27]. Hily et al. [25] showed that the transgene *PPV* CP in the resistant HoneySweet has a high level of methylation and that is re-set in progeny carrying the transgenes [25]. Among the possible co-affected sequences with a low level of methylation, was the CaMV35S promoter [25]. In order to decipher this related silencing with CaMV35S promoter, the hypothesis was a prerequisite that each clone harbors one copy of the virus transgene as a haploid character. Four clones harboring the transgene CP, from the high CP expression parent, clone C2 and the IRSH of the C1738 clone resulted in a silenced CP gene. The associated activities, the chromogenic GUS expression from the C2 parent and the transgene methylation related with the IRSH construct, the present studies provided rational support that the CaMV35S promoter, possibly regulated through the histone modification, did not have any large effect on methylation because it also drives the marker GUS expression (Figure 2). Although the silencing mechanisms that led to a phenotypic variation based onto the knock down of the transgene CP in the 4 hybrid clones is related to an epigenetic pattern [13]. Figure 3a,b show that an epigenetic regulation, based on the methylation of the transgene CP, can occur in the nuclei. There is some evidence that the histone modification led to the inhibition of the activity of the transcription machinery.

As analytical consequences were the methylation of the transgene CP from C2 clone occurred in the four hybrid clones, C-2 × C-1738-4, -7, -28 and -37 hybrids. The real phenomenon is more a “transcription gene silencing” (TGS) rather a post-transcription gene silencing because no RNA is transcribed [27,28]. All four hybrid clones develop the posttranscriptional gene silencing (PTGS) because they harbor the IRSH construct. Referring to Scorza et al.’s results about nuclear-run on assays [7], there was some level of transcribed RNA from nuclei of HoneySweet plum. The level is not so different to that transcribed by the higher CP gene expressor clone C4 [3,7]. Consequently, all four hybrid clones, including C-2 × C-1738-4, -7, -28 and -37 that do not accumulate CP, develop two silencing machinery TGS which functions in nuclei and PTGS which is revealed in cytoplasm. Without any change in the engineered *PPV* CP sequence in plum genome, the methylation status down regulates the CP gene expression in these hybrid plums. PTGS [28] is related to the transcription of the dsRNA from the IRSH that functions in the cytoplasm in order to be diced into siRNA by the dicer-like-proteins [29]. Similarly, to the HoneySweet plum [5,6,7,8,9,16,17,22,23,27], Figure 1 has shown that siRNA accumulated in the C1738 clone most likely recognizing the viral genome and cleaved it as the normal RNAi defense [11,17,27,28,29,30]. In extenso, the Bluebyrd BO70146 × C1738 hybrids (BO70146 × C1738-2, -18, -28) also support the occurrence of the PTGS to trigger any *PPV* RNA restrictively replicating in cytoplasm (Figure 4).

Regardless of the developmental stage of the hybrid clones, the impact of the methylation phenomenon occurring through the chromatin remodeling at the nuclei compartment is obviously extended in the cytoplasm. In order to study the genetic and phenotypic variation related with the engineered transgene, hybrid clones with the Bluebyrd plum as maternal parent and C1738 as paternal progenitor successfully showed that the IRSH, a serendipitous construct resulting from the re-arranged *PPV* CP construct in the plum chromosomes is active (Figure 4). This can happen regardless of the configuration, either Cis or Trans of the CP transgene from clone C2 in the four hybrid (C2xC1738) plum clones. The present study did not precisely identify the orientation but Cis would require them to be on the same chromosome, as in HoneySweet. However, avoiding any speculation, the Cis-element is more powerful. Since Callahan et al. [8] indicated that the known hybrid clones rated from the Mendelian fashion might segregate as a diploid character. This is interesting for breeding any hexaploid *P. domestica* species. Would that really mean that any cross-hybridization with European plum could arguably stand in that way? Unfortunately, the use of the IRSH clone as maternal progenitor gave a poor ratio of hybrid clones. Here, in Europe, the asynchrony flowering of the C-1738 plum clone is among the detrimental cause. Here we showed that the TGS and the PTGS can occur in perennials, both phenomena related with the homology dependent RNA sequencing are suitably active in the two compartment cells. Two major enzymes actively support the silencing mechanism in plum. First, the RNA polymerase II [26,30] that transcribes the dsRNA from the IRSH template in both directions. Secondly, the 24nt siRNA resulting from either the DNA dependent RNA polymerase IV or the tasiRNA (trans-acting RNAi) interacting with the RNA dependent RNA polymerase VI (RDR6) moving as guide to induce the methylation of the homologous DNA sequence [16,17,29].

Expectedly hybrid clones obtained from the cross hybridization between the conventional BlueByrd B70146 plum and C-1738 confirmed the efficient transfer of the IRHS virus gene. Hybrid clones that inherited the IRSH CP successfully silenced the *PPV* genome (Figure 4). These studies compared with the initial use of the IRSH associated with other pieces of PPV CP sequences in the HoneySweet plum [8] elucidate two additive information. First the cross hybridization in any hexaploid hybrid clones is likely inherited with any use of a gene that segregates as a diploid. Second, the epigenetic phenomenon provides evidence about how and where the invading *PPV* genome started to be degraded by the AGO-plant RNasesIII enzyme [26,27,28,30]. Although some definitive experiments were not done, obviously the annealing of the complementary siRNA sequence to the 3′terminal region of *PPV* genome should serve as template to the endonuclease RNase type III [28,29,30]. Avoiding speculation about the following step, the unprotected diced mRNA should be processed according to the siRNA pathways that led to the complete degradation of *PPV* RNA. While the one group of the AGO proteins is preferentially guided by the 24nt-siRNA to induce the transcriptional gene silencing, the second group of AGOs guided by the 21nt-siRNA contribute to the achievement of the PTGS. TGS and PTGS occurring, first in nuclei, inhibit any homologous sequenced RNA [28,30]. This was the scenario in hybrid C-2 × C-1738-4, -7, -28 and -37 that do not express CP gene. Transferred in the cytoplasm, these siRNAs trigger the homologous RNA sequence occurring. In short, *PPV* genome introduced by any means (naturally by viruliferous aphids or artificially by infected graft) [4,5,6,7,15,16,17,18,21,22,23,24,25,29] that starts to be replicated with the viral genome machinery, is specifically triggered by these siRNAs. When systemically spread in the whole plant these siRNAs led to the *PPV* RNA degradation [16,17,22,23,29]. Under conditions of mixed infection including either *Prune dwarf virus* (*PDV*) or *Prunus necrotic ringspot virus*, (*PNRSV*), RNA-silencing derived resistance to *PPV* remains stable. Disregard to the synergistic interactions possibly occurring between viruses, the degradation of *PPV* RNA is related to the homology-dependent RNA silencing [31,32,33].

Although the present study was performed under high containment greenhouse conditions, the robustness of the silencing induced by IRSH *PPV* CP harbored by the HoneySweet plum in natural conditions [18,19,32,33] is one obvious reason for using biotechnology against virus. Long term field trials clearly demonstrated that regardless of different ecological conditions (variable climate, aphid vectors, virus pressure, different strains etc.) in four PPV endemic areas from Poland, Spain, Romania and Czech Republic, the IRSH rearrangement in “HoneySweet” led to the setting up of a high and durable resistance to natural *PPV* infection [18,19,25,32,33].

## 5. Conclusions

The rearranged IRSH construct extracted from the HoneySweet plum is a powerful tool to accurately produce a dsRNA that triggers any homologous dependent sequence co-introduced and, in extenso, the incoming virus infection. Through the epigenetic phenomenon that occurred in progeny, the transgene construct strongly silences either any co-integrated CP gene or any incoming *PPV* RNA in trees with the IRSH construct. These silencing studies gave more accuracy about the two phenomena that occurred, first in nuclei (TGS) and secondly in cytoplasm (PTGS). Subsequently, evidence to degrade the *PPV* RNA from its 3′terminal region strongly supports the sequential cleavage of the virus genome. These studies reflect a successful control strategy about the robust phenotype displayed by HoneySweet plum that is sharing a stable and durable resistance to *PPV* infection in field natural conditions.

## Figures and Tables

**Table 1:** Hybrid clones harboring both a methylated CP transgene and positive GUS.

Cross Hybridization	Rooted Plants	Positive Gus Assays	Methylated cp and Positive Gus
C1738 × C2	3	1/3	0/1
C2 × C1738	60	32/60	4/32
TOTAL	63	33/63	4/33

## Data Availability

Not appropriate.
